# Prevalence and determinants of diarrhoea and acute respiratory infections among children aged under five years in West Africa: evidence from demographic and health surveys

**DOI:** 10.1093/inthealth/ihad046

**Published:** 2023-06-30

**Authors:** Derrick Nyantakyi Owusu, Henry Ofori Duah, Duah Dwomoh, Yakubu Alhassan

**Affiliations:** Research Department, FOCOS Orthopaedic Hospital, P.O.Box KD 779, Accra-Ghana; Department of Population, Family and Reproductive Health, School of Public Health, College of Health Sciences, University of Ghana, Legon, P.O. Box LG 118, Accra, Ghana; College of Nursing, University of Cincinnati, Cincinnati 45221, Ohio; Department of Biostatistics, School of Public Health, College of Health Sciences, University of Ghana, Legon, P.O. Box LG 118, Accra, Ghana; Department of Biostatistics, School of Public Health, College of Health Sciences, University of Ghana, Legon, P.O. Box LG 118, Accra, Ghana

**Keywords:** ARI, children under five, demographic health surveys, diarrhoea

## Abstract

**Background:**

Diarrhoea and pneumonia are the leading causes of morbidity and mortality in children aged <5 y (under five) globally. This study sought to investigate the prevalence and determinants of diarrhoea and acute respiratory infections (ARIs) among children under five in West Africa.

**Methods:**

The most recent demographic and health survey (DHS) standard for 13 West African countries was used in the study. We calculated the prevalence of diarrhoea and ARIs (2 wk prior to the survey) and performed multivariable complex logistic regression analysis to identify possible predictors of diarrhoea and ARIs.

**Results:**

The weighted prevalence of diarrhoea and ARI was 13.7% and 15.9%, respectively. The prevalence of comorbid diarrhoea and ARI was 4.4%. Children aged <2 y (p<0.001), mothers aged <30 y (p<0.003), mothers without formal education (p<0.001), poor households (p<0.001) and poor nutritional status, wasting (p=0.005) and underweight (p<0.001), were the independent predictors of diarrhoea. The independent predictors of ARIs were children with no childhood vaccinations (p=0.002), use of solid fuel in the household (p=0.007), being underweight (p=0.05) and diarrhoea (p<0.001).

**Conclusions:**

The findings imply the need for holistic public health interventions such as increased vaccination coverage, population-based nutritional programmes and campaigns on the use of cleaner cooking fuel targeted at high-risk subgroups in the population to reduce the burden and adverse effects of diarrhoea and ARIs in the West African region.

## Introduction

Diarrhoea and acute respiratory infections (ARIs) remain leading causes of morbidity and mortality in children aged <5 y (under five) worldwide.^1^ According to the most recent WHO estimates, there were approximately 149 million cases of diarrhoea in children under five in 2019, resulting in an estimated 1.4 million deaths. Also, about 42 million cases of ARIs in children under five were reported, resulting in an estimated 630 000 deaths.^1^ The burden of under-five mortality attributable to diarrhoea and pneumonia is higher for children in developing countries compared with developed regions.^2–4^ This may be attributable to the high prevalence of diarrhoea (15.3%) and pneumonia (25.3%) in sub-Saharan Africa (SSA) coupled with the burden of other comorbid diseases and poverty.^5,6^

Numerous interventions have been implemented globally in recent years to combat the prevalence and impact of diarrhoea and ARIs in children. These initiatives have been focused on various fronts, including improved access to clean water and sanitation facilities, vaccination campaigns and community education programmes. These interventions have all played an important role in reducing the burden of diarrhoea and ARIs in children; however, prevalence is still high in the SSA regions.

With just 2 y to the timeline of the Global Action Plan for the Prevention and Control of Pneumonia and Diarrhoea, which aims to reduce death from pneumonia and diarrhoea by 2025, it appears that more focused interventions and research in SSA are required. Some studies have reported that the high prevalence of diarrhoea and ARIs is associated with a myriad of sociodemographic factors, such as the child's nutritional status and age, sanitation, caregiver's education, mother's occupation, wealth quintile, source of drinking water, hand hygiene and Rota virus vaccination, among others.^7–9^

West Africa has a high burden of diarrhoea and ARIs in children under five because of various factors such as poor sanitation, limited access to clean water, inadequate hygiene practices, malnutrition and low vaccination coverage. These factors contribute to the transmission and persistence of infectious agents that cause diarrhoea and ARIs.^10^ Additionally, poverty, weak health systems and limited access to healthcare services make it difficult for affected children to receive timely and appropriate treatment, leading to increased morbidity and mortality.^10^ Therefore, addressing these underlying factors and strengthening healthcare systems is crucial for reducing the burden of diarrhoea and ARIs in children under five in West Africa. Although there is a wealth of information on the prevalence and determinants of diarrhoea and ARIs in specific sites or countries, there is a paucity of literature estimating the prevalence of diarrhoea and ARIs among children under five in West Africa. Hence, this study aims to investigate the prevalence and determinants of diarrhoea and ARIs among children under five in the West African subregion.

## Methods

### Overview of the demographic and health survey

The demographic and health survey (DHS) is a nationally representative cross-sectional survey. The DHS receives technical assistance from International Classification of Functioning, Disability and Health and is predominantly funded by the United States Agency for International Development. Moreover, the survey also receives supplementary support from various international donors, including UNICEF, UNFPA, The World Bank and the Global Fund, among others.

### Study population

Data from DHSs of 13 West African countries were used in this study. The surveys, which are conducted at average intervals of 5 y in every country, are cross-sectional and provide information on health and population characteristics. The most recent DHS for various West African countries was used. The countries involved were Burkina Faso (2010), Benin (2017), Cote d’Ivoire (2011–2012), Ghana (2014), Gambia (2019), Guinea (2018), Liberia (2019), Mali (2018), Nigeria (2018), Niger (2017), Serra Leone (2019), Senegal (2010–2011) and Togo (2013).

The study was conducted in 13 countries in the West Africa subregion, namely, Benin, Burkina Faso, Cote d'Ivoire, Gambia, Ghana, Guinea, Liberia, Mali, Niger, Nigeria, Senegal, Sierra Leone and Togo. These are all developing countries, where child mortality and morbidity rates are among the highest in the world. In total, 132 587 mothers from various West African countries were interviewed for the study. Nigeria had the most participants (23.16%) across the various samples. Burkina Faso had the second highest number, accounting for 10.34% of the overall sample size. Liberia was the country that contributed the least, accounting for 3.96% of the total (Table 1).

**Table 1.** Distribution of unweighted frequencies for West African countries

Country	Unweighted frequency	Unweighted %
Burkina Faso	13 716	10.34
Benin	12 651	9.54
Cote d’Ivoire	7093	5.35
Ghana	5595	4.22
Gambia	7927	5.98
Guinea	7273	5.49
Liberia	5245	3.96
Mali	9275	7.00
Nigeria	30 713	23.16
Niger	11 602	8.75
Serra Leone	9063	6.84
Senegal	5899	4.45
Togo	6535	4.93
**Total**	**132 587**	

### Study variables

Diarrhoea and ARI were the dependent (outcome) variables. Codes 1 and 0 denoted the presence and absence of outcome variables, respectively. ARI prevalence was calculated by asking mothers with children under five whether their child had been ill with a cough, short, quick breaths and difficulty breathing as a result of a chest condition in the 2 wk preceding the DHS.

Diarrhoea prevalence was determined by asking mothers of children born 5 y before the survey if their children experienced diarrhoea episodes in the 2 wk preceding the survey. The child's age (in months), gender, location of residence (rural or urban), wealth quintile, caregiver's age, caregiver's education, water source, toilet facility (improved or unimproved), nutritional status (wasting, stunting and underweight), vaccination status (ever or never vaccinated), breastfeeding status (ever or never breastfed) and have had vitamin A in the last 6 mo (yes or no), were all independent exposure factors.

The household wealth index was determined by considering various household characteristics (such as the source of drinking water, type of toilet, shared facilities, roofing material, wall and floor materials and cooking fuel), as well as household assets (such as ownership of a television, radio, vehicles, bicycles, motorcycles, watches, agricultural land and farm animals). Using factor analysis, weights were assigned to each asset within each household, resulting in a cumulative score. Subsequently, households were ranked based on these cumulative scores. The distribution of the overall wealth score was evaluated, and four specific thresholds were identified to establish quintiles. These distinct thresholds were utilised to categorise household wealth into quintiles, which were denoted as the poorest, poorer, middle, richer and richest categories, respectively, based on their placement within the following percentiles: equal to or below the 20th percentile, above 20% but not exceeding the 40th percentile, above the 40th percentile but not exceeding the 60th percentile, above the 60th percentile but not exceeding the 80th percentile, and above the 80th percentile.

Also, based on WHO criteria, stunting was operationalised as height-for-age Z scores <–2; wasting was defined as weight-for-height Z scores <–2; and underweight was defined as weight-for-age Z scores <–2. For the purpose of this study, each of the three nutritional indicators was coded separately as binary variables, with ‘1’ coded for stunting, wasting and underweight, whereas ‘0’ was used as the code for the absence of stunting, wasting and underweight.

### Data analysis

Prior to data analysis, we performed data cleaning and recoding in Stata version 16 (Stata Corporation, College Station, TX, USA). Next, the data were weighted, allowing us to perform univariate analysis by accounting for survey design. Prior to bivariate and multivariable analysis, complex survey mode was activated using the ‘svyset’ Stata command to enable the adjustment for clusters, stratification and sample weights. This helps to account for possible analytical errors that are embedded within secondary datasets collected using complex sampling designs.^11^ Subsequently, bivariate analyses with the χ^2^ test were conducted to assess the relationship between the selected independent variables and the two dependent variables (diarrhoea and ARI). The strength of the association was estimated using crude and adjusted logistic regression analyses with the ‘logistic’ command. These were performed separately for diarrhoea and ARI.

## Results

### Sample characteristics

The total number of children aged 0–59 mo involved in the study was 75 146. The majority of children were aged 12–35 mo (40.2%). The same age category was high across all countries: Burkina Faso (39.8%), Benin (39.1%), Cote d’Ivoire (42.6%), Ghana (41.2%), Gambia (39.9%), Guinea (38.3%), Liberia (37.6%), Mali (40.2%), Nigeria (40.1%), Niger (38.5%), Serra Leone (39.4%), Senegal (42.0%) and Togo (42.1%). The majority of children sampled in the study were male (50.6%).

The results of nutritional assessments carried out on children in the study were as follows: 31% of children experienced stunting, 20.0% were underweight and 8.4% experienced wasting. Overall, 49.8% of mothers were in the 25–34 y age category. More than one-half (54%) of mothers who participated in the study had no formal education. Regarding individual countries, Nigeria emerged as the country with the highest number of mothers who have had tertiary education, accounting for 8.6%. After Nigeria was Ghana, with 4.5% of mothers who have had tertiary education.

Furthermore, most mothers (43.3%) were in the poor wealth index. For the type of cooking fuel used at home by mothers in West Africa, solid fuel emerged as the most used cooking fuel, accounting for 87.0%. The majority (53.7%) of participants were using unimproved toilet facilities; 70% of participants had access to improved water sources (Table 2).

**Table 2.** Distribution of characteristics across West African countries

	Countries													
	Burkina Faso (%)	Benin (%)	Cote d’Ivoire (%)	Ghana (%)	Gambia (%)	Guinea (%)	Liberia (%)	Mali (%)	Nigeria (%)	Niger (%)	Sierra Leone (%)	Senegal (%)	Togo (%)	Total (%)
Characteristics														
Child age (mo)														
<6	11.4	11.5	11.8	12.1	13.2	12.4	12.0	11.8	10.7	13.2	12.5	11.5	9.2	11.4
6–11	10.7	12.4	12.3	10.6	10.4	9.0	13.4	10.2	10.9	10.2	11.7	11.4	11.4	10.9
12–35	39.8	39.1	42.6	41.2	39.9	38.3	37.6	40.2	40.1	38.5	39.4	42.0	42.1	40.2
36–47	19.4	19.0	17.8	18.7	19.9	21.1	19.3	19.5	19.4	20.5	18.7	18.5	19.5	19.3
48–59	18.4	18.1	15.5	17.4	16.6	19.2	17.7	18.3	18.9	17.6	17.7	16.6	17.7	18.1
Gender														
Male	50.5	50.6	48.9	52.0	51.8	51.7	50.0	50.7	50.8	50.4	50.4	48.9	50.1	50.6
Female	49.5	49.4	51.1	48.0	48.2	48.3	50.0	49.3	49.2	49.6	49.6	51.1	49.9	49.4
Mother’s age (y)														
15–24	28.60	25.30	31.10	20.90	21.40	28.10	34.30	29.20	23.60	27.30	28.70	22.90	21.10	25.20
25–29	26.00	30.90	28.10	25.20	31.20	27.50	25.60	27.70	28.30	27.80	27.60	25.60	28.40	27.80
30–39	36.20	35.60	32.80	43.00	39.00	34.60	31.00	35.40	38.90	36.10	35.40	41.20	40.00	37.80
≥40	9.20	8.20	8.00	11.00	8.40	9.80	9.10	7.7	9.20	8.90	8.30	10.30	10.50	9.20
Mother’s education														
No education	92.3	82.3	81.3	41.4	59.0	84.0	57.2	81.5	49.6	94.3	64.9	80.5	68.9	63.4
Primary/JSH	7.0	15.9	16.5	47.2	29.1	11.8	29.9	17.2	21.1	5.2	28.2	16.0	28.8	19.9
SHS	0.2	0.5	1.1	7.0	7.8	2.0	9.1	0.0	20.6	0.1	4.1	1.0	0.8	11.5
Tertiary	0.6	1.4	1.0	4.5	4.1	2.2	3.9	1.3	8.6	0.4	2.8	2.5	1.6	5.3
Residence														
Urban	17.3	38.9	37.7	45.1	65.7	29.7	53.7	20.7	39.6	13.7	35.3	36.7	36.3	34.9
Rural	82.7	61.1	62.3	54.9	34.3	70.3	46.3	79.3	60.4	86.3	64.7	63.3	63.7	65.1
Wealth index														
Poor	41.80	41.50	47.00	43.0	43.50	44.90	45.80	41.70	43.50	40.0	45.60	46.10	41.10	43.30
Middle	21.7	20.20	19.40	19.60	20.80	19.70	18.70	21.60	20.60	20.50	20.40	18.20	20.10	20.40
Rich	36.5	38.30	33.60	37.40	35.60	35.40	35.40	36.80	35.60	39.50	34.00	35.70	38.80	36.30
Sanitation														
Improved	24.9	27.7	41.2	64.8	62.6	48.0	43.4	53.6	50.6	17.7	50.0	67.3	34.1	46.3
Unimproved	75.1	72.3	58.8	35.2	37.4	52.0	56.6	46.4	49.4	82.3	50.0	32.7	65.9	53.7
Water source														
Improved	74.9	65.7	73.9	84.0	89.4	76.1	80.0	67.0	67.6	65.9	60.4	81.6	60.6	70.0
Unimproved	25.1	34.3	26.1	16.0	10.6	23.9	20.0	33.0	32.4	34.1	39.6	18.4	39.4	30.0
Breastfeeding														
Ever breastfed	98.2	96.1	95.7	98.9	99.0	87.1	98.1	94.1	98.4	98.1	98.1	98.2	98.3	97.6
Never breastfed	1.8	3.9	4.3	1.1	1.0	12.9	1.9	5.1	1.6	1.9	1.9	1.8	1.7	2.4
Vaccination status														
Ever vaccinated	86.7	67.4	84.8	87.7	81.7	58.9	82.2	64.3	67.4	84.0	87.7	82.3	88.2	73.4
Never vaccinated	13.3	32.6	15.2	12.2	12.3	18.3	17.8	35.7	32.6	16.0	12.3	17.7	11.8	26.6
Vitamin A in last 6 mo														
Yes	60.4	48.5	57.6	58.7	49.1	37.2	41.6	61.9	41.4	55.5	62.4	43.4	78.3	48.4
No	39.6	51.5	42.4	41.3	50.9	62.8	58.4	38.1	58.6	44.5	37.6	56.6	21.7	51.6
Type of cooking fuel														
Solid fuel	97.8	97.1	88.8	80.3	98.1	98.5	98.9	99.2	81.0	99.4	99.8	80.6	95.4	87.0
Liquid fuel	0.0	0.1	0.0	0.0	0.2	0.0	0.1	0.0	9.5	0.0	0.0	0.0	0.0	4.9
Cleaner fuel	2.2	2.9	11.2	19.7	1.8	1.5	1.0	0.8	9.6	0.6	0.2	19.4	4.6	8.1
Nutritional status														
Wasting	15.8	5.0	7.8	4.7	5.3	9.1	3.7	8.9	6.9	18.1	5.6	8.0	6.8	8.4
Stunting	34.5	31.6	29.8	17.9	17.0	31.1	28.8	26.7	36.5	43.3	29.1	17.6	26.7	31.7
Underweight	25.7	16.6	14.7	10.8	11.6	16.1	10.3	18.5	21.7	36.2	13.6	14.1	15.7	20.0

Abbreviations: JSH, Junior High School; SHS, Senior High School.

### Prevalence of diarrhoea among children under five in West Africa

**Figure 1.**
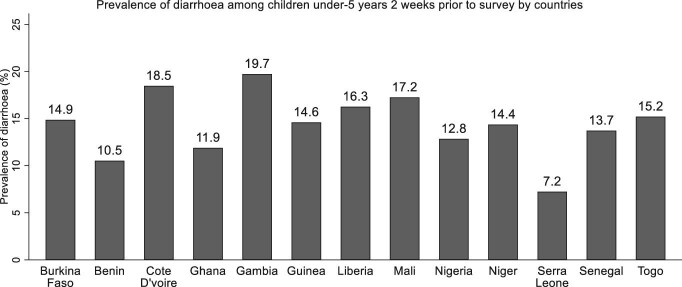
Prevalence of diarrhoea among children under five by country.

The overall prevalence of diarrhoea in West Africa was 13.7%. The prevalence of diarrhoea across West African countries was as follows: Burkina Faso (14.9%), Benin (10.5%), Cote d’Ivoire (18.5%), Ghana (11.9%), Gambia (19.7%), Guinea (14.6%), Liberia (16.3%), Mali (17.2%), Nigeria (12.8%), Niger (14.4%), Serra Leone (7.2%), Senegal (13.7%) and Togo (15.2%) (Figure 1). From the distribution, Gambia was the country with the most children who had diarrhoea 2 wk before the survey, followed by Cote d’Ivoire.

### Prevalence of ARI among children under five in West Africa

**Figure 2.**
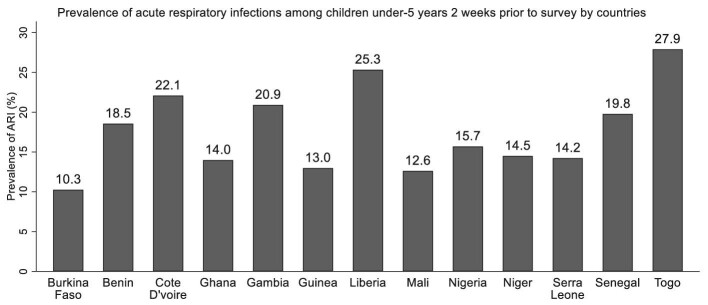
Prevalence of ARI among children under five by country.

The overall prevalence of ARI for West Africa was 15.9%. The prevalence of ARI across West African countries were as follows: Burkina Faso (10.3%), Benin (18.5%), Cote d’Ivoire (22.1%), Ghana (14.0%), Gambia (20.9%), Guinea (13.0%), Liberia (25.3%), Mali (12.6%), Nigeria (15.7%), Niger (14.5%), Serra Leone (14.2%), Senegal (19.8%) and Togo (27.9%) (Figure 2). From the distribution, Togo was the country with the highest burden of ARIs in children 2 wk before the survey, followed by Liberia and Cote d’Ivoire. Furthermore, prevalence for comorbid ARI and diarrhoea was 4.4%.

## Results of bivariate analysis

The following variables had a significant association with diarrhoea prevalence in bivariate analysis: residence, child's age, mother's age, mother's educational level, wealth index, sanitation, water source and nutritional status (Table 3).

**Table 3.** Results of bivariate analysis

	Diarrhoea among children under five				ARI among children under five			
Characteristics	No diarrhoea	Diarrhoea	χ^2^	p	No ARI	ARI	χ^2^	p
**Overall**	**%**	**%**			**%**	**%**		
								
**Country**			470.12	<0.001			1016.41	<0.001
Burkina Faso	85.1	14.9			89.7	10.3		
Benin	89.5	10.5			81.5	18.5		
Cote d’lvoire	81.5	18.5			77.9	22.1		
Ghana	88.1	11.9			86.0	14.0		
Gambia	80.3	19.7			79.1	20.9		
Guinea	85.4	14.6			87.0	13.0		
Liberia	83.7	16.3			74.7	25.3		
Mali	82.8	17.2			87.4	12.6		
Nigeria	87.2	12.8			84.3	15.7		
Niger	85.6	14.4			85.5	14.5		
Serra Leone	92.8	7.2			85.8	14.2		
Senegal	86.3	13.7			80.2	19.8		
Togo	84.8	15.2			72.1	27.9		
**Residence**			285.98	<0.001			36.17	0.01
Urban	88.5	11.5			83.3	16.7		
Rural	85.1	14.9			84.6	15.4		
**Child's age (mo**)			1688.27	<0.001			478.26	<0.001
<6	90.3	9.7			85.5	14.5		
6–11	79.3	20.7			78.7	21.3		
12–35	81.7	18.3			81.3	18.7		
36–47	93.2	9.8			84.6	15.4		
48–59	93.0	7.0			88	12		
**Gender**			4.83	0.120			0.132	0.795
Male	86.1	13.9			84.2	15.8		
Female	86.5	13.5			84.1	15.9		
**Mother’s age (y**)			390.43	<0.001			64.46	<0.001
15–19	80.7	19.3			82.5	17.5		
20–24	84.1	15.9			83.5	16.5		
25–29	86.5	13.5			83.7	16.3		
30–34	87.9	12.1			85.0	15.0		
35–39	87.5	12.5			84.1	15.9		
40–44	87.5	12.5			85.7	14.3		
45–49	88.2	11.8			85.6	14.4		
**Mother’s education**			869.96	<0.001			415.93	<0.001
No education	84.7	15.3			85.9	14.1		
Incomplete primary	83.9	16.1			80.1	19.9		
Complete primary	86.6	13.4			83.2	16.8		
Incomplete secondary	87.0	13.0			81.8	18.2		
Complete secondary	91.6	8.4			82.3	17.7		
Tertiary	93.3	6.7			83.3	16.7		
**Wealth index**			698.12	<0.001			23.28	0.22
Poorest	83.1	16.9			84.4	15.6		
Poorer	84.5	15.5			84.2	15.8		
Middle	86.9	13.1			84.5	15.5		
Richer	87.7	12.3			84.1	15.9		
Richest	90.4	9.6			83.1	16.9		
**Sanitation**			132.86	<0.001			66.43	<0.001
Improved	87.5	12.5			83.2	16.8		
Unimproved	85.3	14.7			84.9	15.1		
**Water source**			133.03	<0.001			0.5480	0.7481
Improved	87.0	13.0			84.2	15.8		
Unimproved	84.6	15.4			84	16		
**Breastfeeding**			0.825	0.5392			16.04	0.008
Ever breastfed	86.3	13.7			84.1	15.9		
Never breastfed	86.9	13.1			86.7	13.3		
**Vaccination status**			7.604	0.09			68.19	<0.001
Ever vaccinated	84.4	15.6			84.1	15.9		
Never vaccinated	85.6	14.4			87.6	12.4		
**Vitamin A in last 6 mo**			3.865	0.292			228.49	<0.001
Yes	86.1	13.9			82.6	17.4		
No	86.5	13.5			85.6	14.4		
**Type of cooking fuel**			662.20	<0.001			34.21	0.07
Liquid fuel	85.4	14.6			84.3	15.7		
Solid fuel	94.6	5.4			84.3	15.7		
Cleaner fuel	91.1	8.9			82.1	17.9		
**Nutritional status Wasting**								
			262.05	<0.001			10.14	0.02
Yes	76.1	20.9			81.8	18.2		
No	86.7	13.3			83.5	16.5		
**Stunting**			221.78	<0.001			14.13	0.01
Yes	83.2	16.8			84.1	15.9		
No	87.3	12.7			82.9	17.1		
**Underweight**			489.71	<0.001			1.676	0.395
Yes	80.2	19.8			82.9	17.1		
No	87.4	12.6			83.4	16.6		

Also, the following variables had a significant association with ARI prevalence: residence, child's age, mother's age, mother's educational level, sanitation, breastfeeding, vaccination status, vitamin A in the last 6 mo, type of cooking fuel and nutritional status (Table 3).

### Multivariable logistic regression estimates of predictors of diarrhoea and ARI among children under five in West Africa

The strength of the association was measured using a multivariable logistic regression model. The model was adjusted for age of child, gender of child, age of mother, place of residence, education level of mother, household wealth index, sanitation, source of drinking water, vaccination status, vitamin A supplement, type of cooking fuel (solid, liquid or clear fuel) and nutritional status.

Compared with children aged 24–59 mo, there was a 57% increased probability of diarrhoea in children aged 6–23 mo (adjusted OR [aOR]=1.57, 95% CI 1.30 to 1.90). Compared with children whose mothers were aged <30 y, those children whose mothers were aged >30 y were 17% less likely to have had diarrhoea 2 wk before the survey (aOR=0.83, 95% CI 0.74 to 0.94). Likewise, children whose mothers had no formal education had a 2.15-fold greater odds of experiencing diarrhoea than those children whose mothers had tertiary education (aOR=2.15, 95% CI 1.65 to 2.80). Compared with children from rich households, those from poor households were 36% more likely to have had diarrhoea 2 wk prior to the survey (aOR=1.36, 95% CI 1.16 to 1.60). Regarding nutritional status, children who experienced wasting (aOR=1.19, 95% CI 1.05 to 1.35) and underweight (aOR=1.36, 95% CI 1.26 to 1.47) had a 19 and 36% increased odds, respectively, of having diarrhoea compared with children who were not classified as experiencing wasting and underweight (Table 4).

**Table 4.** Multivariate logistic regression estimates of predictors of diarrhoea and ARI among children under five in West Africa

Characteristics	Diarrhoea among children under five				ARI among children under five			
	Unadjusted model		Adjusted model		Unadjusted model		Adjusted model	
Residence	cOR [CI]	p	aOR [CI]	p	cOR [CI]	p	aOR [CI]	p
Urban	1.00 [reference]		1.00 [reference]		1.00 [reference]		1.00 [reference]	
Rural	1.34 [1.06, 1.70]	0.014	0.92 [0.77, 1.10]	0.364	0.90 [0.72, 1.14]	0.425	0.90 [0.71, 1.15	0.436
Child's age (mo)								
6–23	1.82 [1.66, 1.96]	<0.001	1.57 [1.30, 1.90]	<0.001	1.32 [1.24, 1.40]	<0.001	1.14 [0.96, 1.34]	0.118
24–59	1.00 [reference]		1.00 [reference]		1.00 [reference]		1.00 [reference]	
Gender								
Male	1.00 [reference]		1.00 [reference]		1.00 [reference]		1.00 [reference]	
Female	0.96 [0.92, 1.00]	0.075	0.99 [0.94, 1.04]	0.931	1.00 [0 .96, 1.05]	0.806	1.01 [0.95, 1.07]	0.679
Mother’s age (y)								
15–24	1.00 [reference]		1.00 [reference]		1 [reference]			
25–29	0.78 [0.73, 0.83]	<0.001	0.92 [0.84, 1.00]	0.078	0.97 [0.90, 1.04]	0.479	1.07 [0.90, 1.27]	0.391
30–39	0.70 [0.64, 0.76]	<0.001	0.84 [0.77, 0.91]	<0.001	0.90 [0.83, 0.97]	0.010	1.05 [0.89, 1.23]	0.538
≥40	0.70 [0.64, 0.77]	<0.001	0.83 [0.74, 0.94]	0.003	0.83 [0.75, 0.91]	<0.001	0.95 [0.76, 1.18]	0.672
Mother’s education								
No education	2.50 [1.93, 3.23]	<0.001	2.15 [1.65, 2.80]	<0.001	0.87 [0.69, 1.10]	0.258	0.79 [0.53, 1.19]	0.271
Primary/JHS	2.07 [1.70, 2.52]	<0.001	1.88 [1.49, 2.38]	<0.001	1.07 [0.89, 1.28]	0.458	1.01 [0.70, 1.46]	0.923
SHS	1.25 [1.02, 1.53]	0.028	1.08 [0.80, 1.46]	0.596	1.07 [0.94, 1.22]	0.280	1.03 [0.76, 1.41]	0.820
Tertiary	1.00 [reference]		1.00 [reference]		1.00 [reference]		1.00 [reference]	
Wealth index								
Poor	1.56 [1.25, 1.94]	<0.001	1.36 [1.16, 1.60]	<0.001	0.95 [0.77, 1.17]	0.636	0.98 [0.74, 1.32]	0.940
Middle	1.21 [1.04, 1.41]	0.011	1.13 [0.99, 1.29]	0.054	0.93 [0.81, 1.07]	0.363	0.96 [0.76, 1.21]	0.747
Rich	1.00 [reference]		1.00 [reference]		1.00 [reference]		1.00 [reference]	
Sanitation								
Improved	1.00 [reference]		1.00 [reference]		1.00 [reference]		1.00 [reference]	
Unimproved	1.20 [1.04, 1.39]	0.012	0.89 [0.79, 1.00]	0.068	0.88 [0.77, 1.00]	0.06	0.93 [0.81, 1.06]	0.287
Water source								
Improved	1.00 [reference]		1.00 [reference]		1.00 [reference]		1.00 [reference]	
Unimproved	1.21 [1.07, 1.37]	0.002	1.01 [0.91, 1.12]	0.737	1.01 [0.87, 1.17]	0.871	1.07 [0.96, 1.19]	0.173
Breastfeeding								
Ever breastfed	1.00 [reference]		1.00 [reference]		1.00 [reference]		1.00 [reference]	
Never breastfed	0.95 [0.77, 1.17]	0.646	1.05 [0.70, 1.59]	0.790	0.81 [0.66, 0.98]	0.039	1.05 [0.72, 1.54]	0.773
Vaccination status								
Ever vaccinated	1.00 [reference]		1.00 [reference]		1.00 [reference]		1.00 [reference]	
Never vaccinated	0.912 [0.76, 1.08]	0.287	0.83 [0.68, 1.99]	0.067	1.75 [0.61, 0.93]	0.008	1.68 [0.53, 0.87]	0.002
Vitamin A in last 6 mo								
Yes	1.00 [reference]		1.00 [reference]		1.00 [reference]		1.00 [reference]	
No	0.96 [0.83, 1.13]	0.689	0.96 [0.81, 1.15]	0.733	0.79 [0.68, 0.94]	0.006	0.98 [0.83, 1.16]	0.877
Type of cooking fuel								
Solid fuel					1.87 [1.68, 2.09]	<0.001	1.36 [1.06, 1.49]	0.007
Liquid fuel					0.85 [0.62, 1.16]	0.325	0.85 [0.58, 1.23]	0.391
Cleaner fuel	1.00 [reference]		1.00 [reference]		1.00 [reference]		1.00 [reference]	
Nutritional status								
Wasting								
Yes	1.71 [1.51, 1.95]	<0.001	1.19 [1.05, 1.35]	0.005	1.11 [0.99, 1.25]	0.057	1.03 [0.92, 1.16]	0.505
No	1.00 [reference]		1.00 [reference]		1.00 [reference]		1.00 [reference]	
Stunting								
Yes	1.39 [1.26, 1.53]	<0.001	1.08 [0.99, 1.19]	0.07	0.92 [0.82, 1.03]		0.91 [0.82, 1.02]	0.131
No	1.00 [reference]		1.00 [reference]		1.00 [reference]		1.00 [reference]	
Underweight								
Yes	1.712 [1.56, 1.86]	<0.001	1.36 [1.26, 1.47]	<0.001	1.03 [0.92, 1.15]	0.584	1.10 [1.01, 1.23]	0.05
No	1.00 [reference]		1.00 [reference]		1.00 [reference]		1.00 [reference]	
Diarrhoea								
No					1.00 [reference]		1.00 [reference]	
Yes					3.16 [2.85, 3.46]	<0.001	2.92 [2.60, 3.29]	<0.001

Abbreviations: aOR, adjusted OR; cOR, crude OR; JSH, Junior High School; SHS, Senior High School.

Also, the model was used to assess predictors of ARI in children under five in the study. The model revealed that children who had never been vaccinated had a 68% increased odds of having an ARI than their counterparts who had received childhood vaccinations (aOR=1.68, 95% CI 0.53 to 0.87). Furthermore, household cooking fuel was found to be a predictor of ARI. Children from households that use solid fuels (e.g. wood, grass, charcoal) had a 36% increased odds of experiencing an ARI (aOR=1.36, 95% CI 1.06 to 1.49) compared with children from households that used cleaner fuel (e.g. liquefied petroleum gas, electricity). Similar to diarrhoea, children who were underweight had a 10% increased odds of being diagnosed with an ARI (aOR=1.10, 95% CI 1.01 to 1.23). Interestingly, children who had been diagnosed with diarrhoea at the time of the study had a 2.92-fold greater odds of being diagnosed with an ARI than those children without diarrhoea (aOR=2.92, 95% CI 2.60 to 3.29) (Table 4).

## Discussion

In this study, researchers investigated the prevalence and determinants of diarrhoea and ARIs in children under five in West Africa. It was observed that the prevalence of diarrhoea among children under five in West Africa was 13.7%. The prevalence of diarrhoea in the current study was marginally below the reported prevalence of 15.3% in a study conducted in SSA.^8^ Contrarily, the current prevalence is above that of other countries in different regions, such as India (5%),^12^ Vietnam (11%)^13^ and Mesoamerica (13%).^14^

Furthermore, the prevalence of ARI in this study was 15.9%, which was below the prevalence rate reported by studies conducted in Australia (19.9%) and SSA (25.3%).^6,15^ Variations in prevalence could be attributable to differences in the environment and infrastructure, such as improved water sources, the presence of improved toilet facilities, and improved waste disposal methods.^16^ This variation could also be influenced by the timeframe for recalling symptoms. The timeframe for recalling symptoms in this study was 2 wk before the survey, whereas for the Australian study it was 4 wk. Finally, the current study showed that about 4.4% of children had both diarrhoea and ARI 2 wk prior to the survey. This prevalence is twice the prevalence of having both diarrhoea and ARI reported by a study conducted in East Africa.^8^ The comorbid patterns of these conditions highlight the seriousness with which the individual conditions must be prevented and treated to avert the potential negative impact of the concurrent conditions in children under five.

Children aged <2 y (i.e. 6–23 mo) had a significantly higher risk of diarrhoea and ARIs than older children. This could be attributable to the lower immunity in children under five. Although the risk factors of ARI and diarrhoea are complex and cannot be attributed solely to the child's age, it appears that this is a significant contributor. This finding is consistent with previous studies that reported a higher likelihood of ARIs and diarrhoea among children aged <36 mo.^17,18^

Furthermore, approximately one-half of the mothers in the survey had no formal education. This finding is not surprising because a UNESCO assessment indicated that Africa has the greatest rates of school exclusion, particularly among women.^19^ Additionally, it has been demonstrated that the increased economic burden in subregions of Africa translates into families' incapacity to provide formal education for their children. Also, cultural factors may explain the high prevalence of uneducated women. Until recently, most women in Africa's subregions were expected to mainly undertake domestic chores, that is, women were supposed to stay at home and handle all the domestic responsibilities. The same cultural practices in Africa encouraged early marriages, depriving women of formal education.^20,21^ The level of education of mothers was linked to a higher prevalence of diarrhoea in their children. Diarrhoea was twice as common in children whose mothers had no formal education. This is not surprising considering that education enables women to be well informed about how to find and utilise appropriate child health information.^22,23^

The current study also found that children in poor households had a higher probability of experiencing diarrhoea compared with children in rich households. In general, children in poor households may face a level of social and health inequalities that can predispose them to preventable health conditions. Poor households are likely to experience inconsistent nutritional supplies and live in unhygienic neighbourhoods, which increases the risk of exposure to the microbial agents that cause diarrhoea. This finding conforms with those of previous studies conducted in Kenya and Ethiopia.^8,24^

Also, the current study showed that children who were malnourished (i.e. experiencing wasting and underweight) were likely to experience diarrhoea and ARIs. The plausible explanation for this is that malnutrition among children generally denies them the essential micronutrients needed to combat childhood-related diseases such as diarrhoea. Malnutrition enables diarrhoeal infections to occur more frequently and for longer periods, with about a 38% increase in frequency and a 73% increase in length accounting for a doubling of the diarrhoea burden in malnourished children.^14^

The study also found that the use of solid fuel for cooking in households was associated with a higher odds of ARIs in children. This finding is congruent with other studies that have revealed that solid fuel produces a lot of smoke which causes household air pollution (HAP) and renders children vulnerable to a higher probability of respiratory infection.^25,26^ HAP from the burning of solid fuel predisposes the entire home to elevated carbon monoxide (CO) and particulate matter (PM_2.5_) levels, which can lead to an increased risk of respiratory diseases. The detrimental effects of HAP appear to disproportionately affect vulnerable groups such as children. Air pollution from the burning of solid fuels primarily affects the respiratory system, resulting in a variety of acute and chronic symptoms. Respiratory side effects might range from minor symptoms and changes to life-threatening illnesses and even death. Households may benefit from improved cooking fuel. Those that cannot make the transition to clean fuel can use improved cooking stoves, which are associated with low HAP, to reduce the risk of respiratory diseases.^27^

Interestingly, the current study observed an association between diarrhoea and ARI. Children under five in West Africa who had diarrhoea were more likely to have an ARI than those children who had no diarrhoea. One plausible explanation for this is that diarrhoea appears to dehydrate children and deprive them of important micronutrients that aid in immune strengthening. Thus, impaired immunity predisposes them to infections such as ARIs. It is also plausible that both ARI and diarrhoea are symptoms of another underlying condition. Given the cross-sectional nature of the study, we were unable to describe the order of occurrence of the two conditions and no causal inference can be made regarding the association between diarrhoea and ARI. This finding is consistent with earlier research concerning the impact of diarrhoea on children under five.^5,1^^4^

Moreover, the study showed that children who have never had a vaccination were at a higher risk of experiencing an ARI compared with children who had received vaccinations. This finding is congruent with other studies in Asia and Africa that have revealed an association between vaccination status and ARI.^28,29^ In those studies, the association was plausible because vaccines help children to build immunity against infections. Pneumococcal vaccines, for example, help to reduce the risk of respiratory infections.

### Strengths and limitations

The strengths of the current study relate to the use of a large amount of nationally representative data from 13 countries in West Africa. The large dataset implies that the study is adequately powered. We also adjusted for sampling weight, primary sampling units and strata to ensure correct estimates of standard errors. However, for the 13 countries studied here, the most recent DHS was used, and several of these were conducted in different years. Moreover, the DHS is cross-sectional in nature, hence no causal inferences can be made from the observed associations.

### Conclusions

The current study sought to investigate the prevalence and determinants of diarrhoea and ARIs in children under five across 13 different countries in the West African subregion. The overall prevalence of diarrhoea and ARI was 13.7% and 15.9%, respectively. The comorbid burden of the two conditions was 4.4%. Children aged <2 y, mothers aged <30 y, mothers without a formal education, poor households and poor nutritional status (experiencing wasting and underweight), were the independent predictors of diarrhoea. The independent predictors of ARI were children with no childhood vaccinations, use of solid fuel in the household, being underweight and having diarrhoea. These findings imply a need for holistic public health interventions, such as increased vaccination coverage, community-based nutritional programmes and campaigns promoting the use of cleaner cooking fuel, targeted at high-risk subgroups in the population to reduce the burden and adverse effects of diarrhoea and ARIs in the West Africa subregion.

## Data Availability

Data used for the study are freely available after a simple online request at https://dhsprogram.com/data/dataset_admin/index.cfm.
